# Development of prediction model for osteoporotic vertebral compression fracture screening without using clinical risk factors, compared with FRAX and other previous models

**DOI:** 10.1007/s11657-021-00957-y

**Published:** 2021-06-03

**Authors:** Pongsthorn Chanplakorn, Thamrong Lertudomphonwanit, Nuttorn Daraphongsataporn, Chanika Sritara, Suphaneewan Jaovisidha, Paphon Sa-Ngasoongsong

**Affiliations:** 1 Department of Orthopedics, Faculty of Medicine Ramathibodi Hospital, Mahidol University, 270, Rama VI Road, Ratchathewi, Bangkok, 10400 Thailand; 2 Department of Orthopaedic Surgery, Nan Hospital, Nan, Thailand; 3 Department of Diagnostic and Therapeutic Radiology, Faculty of Medicine Ramathibodi Hospital, Mahidol University, Bangkok, Thailand

**Keywords:** Osteoporosis, Vertebral fracture, Predictive model, Bone mass density, Vertebral fracture assessment

## Abstract

***Summary*:**

This study developed a prediction model to assess the need for asymptomatic osteoporotic vertebral compression fracture (OVCF) screening in women without using clinical risk factors. Our results demonstrated that the combination of age, height loss, and femoral neck T-score can predict OVCF comparable to previous models, including FRAX.

**Purpose:**

Osteoporotic vertebral compression fracture (OVCF) is a major fracture in osteoporosis patients. Early detection of OVCF can reduce the risk of subsequent fractures and death. Many existing diagnostic tools can screen for the risk of osteoporotic fracture but none aim to identify OVCF. The objective of this research is to study a predictive model for capturing OVCF and compare it with previous models.

**Methods:**

A retrospective review was conducted that included women aged ≥ 50 years who underwent dual-energy X-ray absorptiometry and vertebral fracture screening between 2012 and 2019. The data included age, height, weight, history of height loss (HHL), and bone mass density (BMD). Receiver operating characteristic analysis and univariate and multivariate logistic regression were performed. The predictive OVCF model was formulated, and the result was compared to other models.

**Results:**

A total of 617 women, a 179 of which had OVCFs, were eligible for analysis. Multivariate regression analysis showed age > 65, height loss > 1.5 cm, and femoral neck T-score < -1.7 as independent risk factors for OVCF. This model revealed comparable performance with FRAX. The model without BMD revealed superior performance to FRAX and other standard osteoporosis assessment models.

**Conclusions:**

BMD and vertebral fracture screening should be eligible for individual women age > 65 years with an HHL more than 1.5 cm, regardless of BMD. Vertebral fracture assessment should be additionally conducted on these women with a femoral neck T-score less than -1.7.

## Introduction

Osteoporosis is a disease that causes a global burden due to exponential increases in aging populations worldwide. Preventive measures and early intervention before the occurrence of major fractures that result in poor quality of life could reduce the disease burden, treatment costs, morbidity, and mortality [1, 2]. In a 2000–2001 nationwide survey, the age-adjusted prevalence of osteoporosis, based on dual-energy X-ray absorptiometry (DXA), in Thai women in ages ranging from 40–80 years was 13.6% for femoral neck and 19.8% for lumbar spine [3]. Results from a previous study also revealed an increased incidence of vertebral fractures with advancing age [4]. According to estimates, the population aged 60 years and over in Thailand will increase from 13 million in 2020 to nearly 24 million by 2050 [5]. Therefore, the osteoporotic vertebral fracture could be a major health problem that requires health professionals to have awareness on the prevention, diagnosis, and management of the condition.

In the management of osteoporosis, osteoporotic vertebral compression fracture (OVCF) is a major concern because it increases the risk of subsequent vertebral and non-vertebral fractures, including the risk of death [6–8]. A meta-analysis revealed that the presence of vertebral fracture increases the relative risk of subsequent hip fracture by 2.3 times and increases to 4.4 times the risk for subsequent vertebral fracture [9]. However, the diagnosis of the OVCF is challenging because only one-fourth of OVCF patients is symptomatic [10], even though early detection of OVCF is crucial because it may prevent subsequent fracture by providing early treatment of osteoporosis. The development of effective screening criteria is therefore very necessary. DXA remains a standard diagnostic tool for osteoporosis. The densitometric lateral spine imaging, called vertebral fracture assessment (VFA), can efficiently and quickly be performed at the time of a bone density test and can accurately detect moderate-to-severe OVCFs. Therefore, the International Society for Clinical Densitometry (ISCD) recommended a VFA for those with a femoral neck of T score < -1 combined with one of the following criteria: (a) women aged ≥ 70 years, (b) men aged ≥ 80 years, (c) history of height loss (HHL) over 4 cm, (d) self-reported but undocumented vertebral fracture, and (e) glucocorticoid intake of more than 5 mg of prednisolone or equivalent per day for more than 3 months [11, 12]. Notably, the recommendation for measuring axial bone density by DXA is already recommended in very high-risk populations, such as females aged over 65 years, patients with prolonged glucocorticoid administration, patients with a history of fraternal or maternal hip fracture, and menopausal women with decreased height of at least 4 cm [2]. However, the effectiveness of this recommendation is still controversial and might not be able to detect early OVCF in a majority of the population. The DXA machine is also not widely available in many areas, so a simple screening tool to identify the population at risk for OVCF without requiring a DXA measurement is ideal.

Many diagnostic screening tools have been introduced to identify risk of osteoporotic fracture. A fracture risk assessment tool (FRAX) was introduced in 2008 to estimate the individualized 10-year probability of hip and major osteoporotic fracture [13]. Although FRAX could predict the hip fracture and shows the highest gradient risk when bone mass density (BMD) is co-entered, the ability to identify the risk of other fracture rather than hip fracture is still limited, especially for OVCF. In addition, some of the clinical risk factors, glucocorticoid in particular, are not accounted for in the dose response [14]. In 2001, the Osteoporosis Self-Assessment Tool for Asians (OSTA) index was introduced to identify the population at risk for osteoporosis and to justify the BMD assessment, but the score is complex and must be adjusted based on ethnicity [15]. More importantly, the OSTA index is not designed for assessing OVCF risk. In 2004, the Khon Kaen Osteoporosis Study (KKOS) introduced the clinical risk index for predicting osteoporosis in Thai women. The KKOS index uses age and weight to individually raise suspicions osteoporosis based on patient BMD [16]. This model also does not aim to identify the individual at OVCF risk. Additionally, the occurrence of OVCF in individuals with BMD in the osteopenic range has been reported [12, 17, 18]. The objective of the current research is to study a predictive model to capture OVCF with and without using the DXA and to then compare the results to previous models.

## Materials and methods

Institutional Review Board approval was obtained at our university medical center (protocol number MURA 2019/1247). A retrospective review was conducted using a patient records database, and the study included women aged ≥ 50 years who came to the Orthopedics Clinic at Ramathibodi Hospital between January 1, 2012, and December 31, 2019, and who underwent DXA and VFA for osteoporosis screening. The exclusion criteria were patients with a history of spinal fracture resulting from high-energy trauma, prior osteoporosis treatment, secondary osteoporosis, and spinal diseases (e.g., spinal infection and malignancy). Patients who had prior spinal surgery or previous hip fracture were also excluded.

The DXA was performed at the lumbar spine in the antero-posterior (AP) view and at the hip in true AP view of the non-dominant and non-fracture side, according to the recommendations from Thai Osteoporotic Foundation (TOPF) [2], in patients with a risk factor for osteoporosis. The indications for DXA examination at our general orthopedic clinic were as follows: (a) early menopause before 45 years, including bilateral oophorectomy; (b) prolonged glucocorticoid administration (daily prednisolone 7.5 mg or equivalent for at least 3 consecutive months); (c) history of paternal hip fracture; (d) menopausal women with decreased height of at least 4 cm; (e) radiographic osteopenia and/or vertebral deformity by X-ray; (f) history of fracture from low-energy injury; and (g) FRAX assessment for risk of fracture, specific to Thai population, without bone mineral density (BMD) showing a 10-year probability of fracture in intermediate risk (≥ 5.0 to < 7.5) or rated in the intermediate group by OSTA score (-1 to -4) or KKOS score (probability of osteoporosis 21–79%). The VFA was requested in every case at the time the DXA test was performed.

In our protocol, the BMD measurement was examined at the lumbar spine (L1–L4), femoral neck (FN), and total hip (TH) using a Discovery™ DXA system (Hologic, Bedford, MA, USA). The T-score was calculated according to the Asian reference database. The OVCF diagnosis from VFA was performed by DXA according to the semi-quantitative technique of Genant using an experienced musculoskeletal radiologist [19, 20]. The DXA and VFA results were reviewed by the picture archiving and communication system (PACS) at our hospital. Age, height, weight, and HHL data were retrieved from an electronic medical records database. HHL was defined as the difference between the current height and the last recorded height in the past 3 years. FRAX fracture risk assessment for 10-year major osteoporosis fracture and hip fracture was calculated for all participants using an ethnic-specific Thailand FRAX™ model (https://www.sheffield.ac.uk/FRAX/tool.aspx?country=57) [21], based on the clinical characteristics, without clinical risk factors and BMD. In addition, the OSTA index for Thai ethnicity, without clinical risk factors, was calculated using the formula (body weight [kg] – age [year]) × 0.2, as index weight was 2 for body weight every 10 kg and index weight was -2 for age every 10 years, according to the original report [15]. The KKOS score was calculated based on age and body weight, as described in the original report [16]. The ISCD for OVCF risk assessment was calculated based on age and HHL more than 4 cm in an individual subject with a FN T-score of less than -1.0 [12]. The clinical risk factors were not included in the present study, allowing us to homogenize the score and reduce the error for OVCF risk estimation in each model.

### Statistical analysis

Statistical analysis was performed using STATA SE version 16.0 (StataCorp, College Station, Texas, USA). Categorical data were analyzed by the Chi-square test, and continuous variables were analyzed using a T-test or Wilcoxon rank-sum test for data as appropriate. The receiver operating characteristic (ROC) curve with Youden index was used to determine the cutoff value for each variable. The correlation coefficient was used to confirm the correlation of each variable according to age. Univariate analysis was used to determine the factors that showed significant difference between the fracture and non-fracture groups. A stepwise logistic regression analysis was performed and included variables with a p value > 0.05 from the univariate analysis. The performance tests were calculated by the Chi-square test to determine the sensitivity, specificity, and likelihood ratio (including positive and negative) predictive value in each model. p Value was < 0.05, indicating statistical significance.

## Results

### Demographic data

**Fig. 1:**
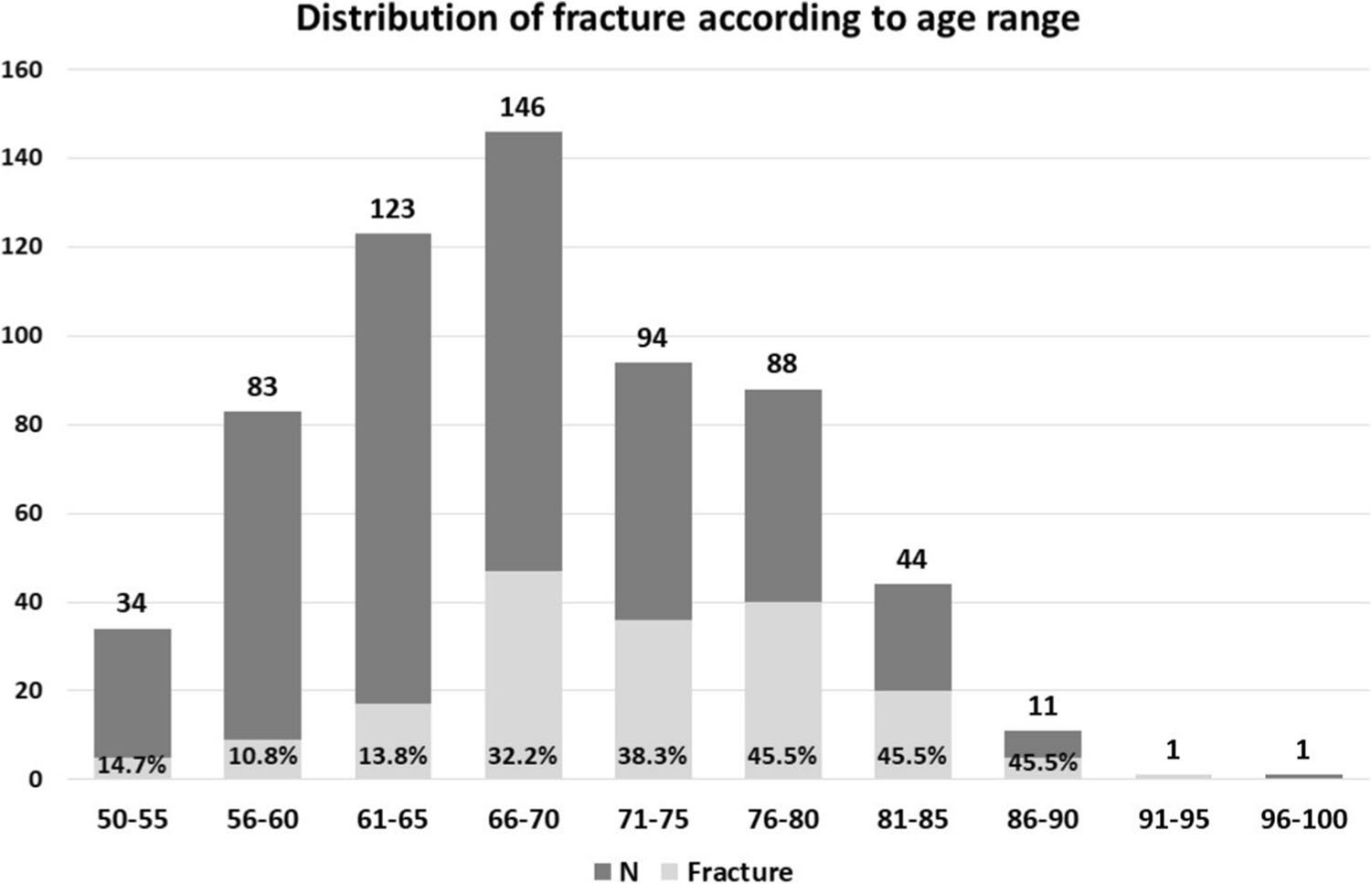
The prevalence of VFF according to age range; a number of subjects (N) according to age and the prevalence of VFF, in percentages, are both shown in the bar graph

During the study period, 617 women were eligible for the analysis. The average age was 68.52 ± 8.56 years. The OVCF was identified in 179 women (29%). On these, the OVCF was identified with an additional level in 43 women, 2 levels in 23 women, 3 levels in 12 women, and 4 levels in 6 women. The most common OVCF occurred at L1, T12, L2, and T11, respectively. The prevalence of OVCF was highest in the age range of 76–90 years (45.5%), followed by 71–75 years (38.3%), 66–70 years (32.2%), and 61–65 years (13.8%). The prevalence of OVCF for age less than 65 years was 11.8%, compared to 38.7% for age more than 65 years (Fig. 1). The OVCF group was significantly higher age-wise than the non-OVCF group; meanwhile, height was lower in the OVCF group, and the HHL was greater in the OVCF group. Regarding BMD, the T-score for all sites was lower in the OVCF group compared to the non-OVCF group (Table 1). The steroid used in our sample was only 2 participants, 1 in the non-OVCF group, and another in the OVCF group.

**Table 1:** Demographic data between fracture (VFF) and non-fracture group

Characteristics		Whole cohort (n = 617)	No fracture (n = 438)	Vertebral fracture (n = 179)	p Value		Youden criterion*
Value displayed in mean ± SD^a^							
Age (year)	68.52 ± 8.56		66.97 ± 8.32	72.31 ± 7.98		< 0.01	*65.00*
Height (cm)	151.55 ± 5.83		152.13 ± 5.80	150.14 ± 5.67		< 0.01	*152.00*
Weight (kg)	56.17 ± 9.91		56.29 ± 9.87	55.89 ± 10.03		0.654	58.80
BMI (kg/m^2^)	24.47 ± 4.18		24.35 ± 4.22	24.76 ± 4.08		0.258	24.49
Value displayed in median (min, max)^b^							
History of height loss (cm)		1.0 (0, 9)	0.5 (0, 6)	1.5 (0, 9)	< 0.01		*1.50*
BMD T-score L1–L4		-1.7 (-5.6, 3.1)	-1.6 (-5.1, 2.6)	-1.9 (-5.6, 3.1)	0.017		*-1.80*
BMD T-score femoral neck		-2.0 (-4.9, 1.4)	-1.9 (-4.4, 1.4)	-2.2 (-4.9, 0.4)	< 0.01		*-1.70*
BMD T-score total hip		-0.9 (-4.3, 2.7)	-0.8 (-4.3, 2.7)	-1.0 (-4.0, 1.4)	< 0.01		*-0.50*
Steroid usage n (%)		2 (100%)	1(0.2%)	1 (0.6%)	0.513^+^		N/A

^a^Data was in normal distribution: the statistical difference was calculated by T-test

^b^Data was not in normal distribution: the statistical difference was calculated by Wilcoxon rank-sum test

^+^Chi-square test

^*^Calculated from ROC analysis

Italics: significant level for area under the curve (AUC) = 0.05, *N/A* not applicable

### The ROC curve and correlation analysis

The ROC curve with Youden index was used to determine the cutoff value in each continuous data that revealed statistical significance between the OVCF and non-OVCF groups. The results are illustrated in Table 1. The cutoff value was 65 years for age (p value < 0.001, AUC 0.69), 152 cm for height (p value < 0.001, AUC 0.60), and 1.5 cm for HHL (p value < 0.001, AUC 0.67). Regarding the BMD, the cutoff value of the T-score differed based on the site of measurement. The cutoff T-score for the L1–L4 area was -1.8 (p value = 0.014, AUC 0.56), -1.7 for femoral neck (p value < 0.001, AUC 0.63), and -0.5 for TH (p value < 0.001, AUC 0.61). Then the participants were divided into groups according to the cutoff point to facilitate further analysis.

The Pearson correlation coefficient was performed to estimate the degree of correlation between age and height, which showed an inverse correlation (R2 = -3.5, p < 0.0001). Spearman’s coefficient of rank was performed to determine the correlation between age and other variables. The results revealed positive but not strong correlation with HHL (R2 = 0.13, p = 0.0015). Regarding BMD, the T-score for any site revealed a negative correlation with age, but there was no significant correlation between age and the L2–L4 area T-score (p = 0.45).

### Univariate and logistic regression analysis

The univariate analysis was performed after the groups were separated by Youden index. The univariate analysis identified 5 factors significantly associated with increased OVCF risk: age > 65 years, height < 152 cm, height loss > 1.5 cm, T-score of < -2.0 at L1–L4 vertebrae, < -1.7 at femoral neck, and < -0.8 at TH. Weight, body mass index, and steroid usage showed no significant association with the occurrence of OVCF in our data. The p value and relative risk (RR) at a 95% confidence interval (CI) are illustrated in Table 2. Results from the logistic regression analysis revealed 3 independent factors associated with OVCF: age > 65 years, HHL > 1.5 cm, and T-score at femoral neck > -1.7. The overall model fit showed significance with p < 0.0001 and Nagelkerke R^2^ = 0.212; the goodness-of-fit test for this model, revealed by Hosmer and Lemeshow, demonstrated significance at p = 0.975 and Chi-square test = 0.8367 with an AUC of 0.742. The overall fit for the model without using BMD showed significance with p < 0.0001 and Nagelkerke R^2^ = 0.192 with an AUC of 0.716 (Table 3).

**Table 2:** Relative risk of VFF according to each factor

Characteristics	Relative risk (95% CI)	p Value
Age > 65 years	4.50 (2.83–7.15)	< *0.01*
Height < 152 cm	2.25 (1.57–3.22)	< *0.01*
Weight < 59 kg	1.19 (0.83–1.73)	0.345
BMI > 24.49 kg/m^2^	1.34 (0.95–1.90)	0.099
History of height loss > 1.5 cm	3.47 (2.38–5.07)	< *0.01*
BMD T score L1–L4 < -1.8	1.50 (1.05–2.13)	*0.024*
BMD T score femoral neck < -1.7	2.74 (1.83–4.12)	< *0.01*
BMD T-score total hip < -0.5	2.34 (1.55–3.53)	< *0.01*
Steroid use	2.46 (0.15–39.47)	0.526

Relative risk was calculated from univariate analysis

*CI* confidence interval, italics variables with p value > 0.05, included in logistic regression analysis

**Table 3:** Independent risk factor associated with VFF

Characteristics	Relative risk (95% CI)	p Value	Score*
Model 1: with BMD (AUC 0.7414)			
Age > 65 years	3.93 (2.38–6.51)	< 0.01	4
History of height loss > 1.5 cm	3.05 (2.05–4.57)	< 0.01	3
BMD T-score femoral neck < -1.7	2.15 (1.39–3.33)	< 0.01	2
Model 2: without BMD (AUC 0.7160)			
Age > 65 years	4.24 (2.58–6.99)	< 0.01	4
History of height loss > 1.5 cm	3.14 (2.12–4.65)	< 0.01	3

Relative risk and p value were calculated from stepwise logistic regression analysis

Score was adjusted from relative risk

*CI* confidence interval, *AUC* area under the curve according to the model

“Score*” used to described that Score was adjusted from the relative risk

### Performance analysis

Using the factors identified from logistic regression analysis, the data was re-grouped and calculated to determine the sensitivity, specificity, and positive and negative predictive value that would predict the OVCF. The results for other models were calculated, for comparison, using our database on the cutoff recommended from the original report (Table 4). In our model with the BMD assessment, having all parameters included revealed the highest specificity, and the HHL > 1.5 cm plus femoral neck T-score < -1.7 revealed the highest sensitivity and odd ratio. The negative predictive value in our model was 80% and over. The highest AUC was demonstrated in the group with age > 65 years and HHL > 1.5 cm. Compared to the FRAX model, our model revealed comparable performance. Regarding the model without BMD, the highest sensitivity was revealed in HHL > 1.5 cm, followed by age > 65 years. The group that included all parameters revealed the highest specificity. The negative predictive value was over 80% in this model. Compared to other models, the present model revealed superior performance to the FRAX without BMD, KKOS, and OSTA models. Our model had comparable performance to the ISCD 2019 statement based on the AUC but revealed higher specificity. Table 5 shows the performance of the present model without BMD according to age groups. The negative predictive value was highest in age group < 65 years with HHL < 1.5 cm. The sensitivity, specificity, and AUC when performed in a 5-year age increment starting from 65 years revealed little difference among groups.

**Table 4:** Performance of the predictive model for predicting VFF and comparison with previous models

	Cutoff	Sensitivity	Specificity	AUC	PPV	NPV	LR +	LR-	Odd ratio
Model 1 (with BMD)									
HHL > 1.5 cm + BMD FN < -1.7	5	83%	52%	0.67	42%	88%	1.73	0.33	5.23
Age > 65 years + HHL > 1.5 cm	7	79%	57%	0.68	43%	87%	1.82	0.38	4.84
Age > 65 years + BMD FN < -1.7	6	51%	81%	0.66	52%	80%	2.65	0.61	4.36
All parameters	9	43%	86%	0.65	56%	78%	3.09	0.66	4.67
FRAX MOF with BMD	10	36%	85%	0.6	50%	76%	2.41	0.76	3.19
FRAX HF with BMD	3	51%	73%	0.62	44%	79%	1.90	0.67	2.83
Model 2 (without BMD)									
HHL > 1.5 cm	3	93%	32%	0.63	36%	92%	1.38	0.2	6.68
Age > 65 years	4	83%	52%	0.66	42%	88%	1.73	0.33	5.23
All parameters	7	51%	81%	0.66	52%	80%	2.65	0.61	4.36
FRAX MOF without BMD	10.00	12%	95%	0.53	48%	72%	2.23	0.93	2.4
FRAX HF without BMD	3.00	30%	85%	0.57	44%	75%	1.94	0.83	2.33
OSTA	-1.00	76%	42%	0.59	35%	81%	1.29	0.58	2.21
	-4.00	30%	82%	0.56	41%	74%	1.68	0.85	1.98
KKOS	-1.00	72%	46%	0.58	35%	80%	1.31	0.63	2.09
ISCD 2019 model with BMD	none	60%	68%	0.64	44%	81%	1.89	0.58	3.24

Cutoff value was calculated from sum of individual risk scores in Table 3 and according to original values reported for other models

*AUC* area under the curve, *PPV* positive predictive value, *NPV* negative predictive value, *LR* + positive likelihood ratio, *LR-* negative likelihood ratio

**Table 5:** Performance of predictive model without BMD according to age

	Cutoff	Sensitivity	Specificity	AUC	PPV	NPV	LR +	LR-
Model 2 (without BMD)								
Age < 65 + HHL > 1.5	3	41%	87%	0.64	28%	92%	3.15	0.68
Age > 65 + HHL > 1.5	7	51%	81%	0.66	52%	80%	2.65	0.61
Age 65–70 + HHL > 1.5	7	32%	81%	0.57	42%	74%	1.74	0.83
Age 71–75 + HHL > 1.5	7	51%	79%	0.65	62%	71%	2.43	0.62
Age 76–80 + HHL > 1.5	7	49%	73%	0.61	58%	65%	1.80	0.70
Age > 80 + HHL > 1.5	7	68%	79%	0.74	56%	78%	3.30	0.40

Cutoff value was calculated from sum of individual risk scores in Table 3 and according to original values reported for other models

*AUC* area under the curve, *PPV* positive predictive value, *NPV* negative predictive value, *LR* + positive likelihood ratio, *LR-* negative likelihood ratio

## Discussion

In the management of osteoporosis, OVCF is a major concern because it increases the risk of subsequent vertebral and non-vertebral fractures, including the risk of death [6–8]. However, diagnosing OVCF is challenging since most OVCF patients are asymptomatic [10], even though early detection of OVCF is crucial because it may prevent subsequent fracture by identifying the need for osteoporosis treatment. The results from the present study revealed a significant difference between age, height, HHL, and BMD of both TH, FN, and L1–L4 T-scores between OVCF and non-OVCF groups. The height, HHL, and FN T-score showed inverse correlation to age, but the L1–L4 T-score did not reveal a statistical correlation. Age more than 65 years, HHL more than 1.5 cm, and the FN T-score less than -1.7 were significant predictors of OVCF.

In this study, the clinical risk factors were not included in our predictive model since we aimed to compare all OVCF predictive models by weighing all clinical risk factors. Evidence regarding the clinical risk factors has been reported particularly in the FRAX model. Kanis et al. studied the gradient of risk among age between hip fracture and major osteoporotic fracture in the FRAX model. The study revealed that including the clinical risks to BMD, compared to BMD alone, barely increases the risk of other osteoporotic fracture, in contrast to hip fracture, for which an increased risk has been demonstrated [14]. Lconaru revealed that, in the FRAX model, the clinical risk factors described in FRAX are not consistent risks in each fracture site. The study shows that prior osteoporotic fracture, age, smoking, and TH BMD remain independent predictors for hip fractures, whereas osteoporosis, age, prior osteoporotic fracture, glucocorticoids used, and spine BMD are independent predictors for OVCF [22]. The results from two such studies could imply that the clinical risk factors provided in the FRAX model have little influence on OVCF prediction, except glucocorticoid use being an independent risk factor for spine fractures only [22, 23]. In our analysis, the prior history of fracture was not included. In addition, the glucocorticoid usage was not shown to be a statistically significant factor for OVCF risk (Table 2), and spine (L1–L4) BMD was also not a significant predictor of OVCF in multivariate analysis (Table 3).

The performance of the FRAX model in predicting OVCF is illustrated in Table 4. In this model, the prediction for the 10-year probability of major osteoporotic fracture was 10% (FRAX-MOF), compared to the hip fracture at 3% (FRAX-HF); FRAX-MOF showed higher sensitivity compared to FRAX-HF (48% vs. 32%), revealed lower specificity (74% vs. 86%), and showed slightly higher AUC (0.61 vs. 0.59). When we compared the FRAX model to our predictive model with BMD and included all risk factors, the results revealed a comparable performance with sensitivity of 44%, specificity of 85%, and an AUC of 0.64. Therefore, the osteoporosis treatment should be provided at this stage to prevent OVCF. When we performed analysis without BMD, the FRAX model showed much lower sensitivity than our predictive model without BMD when all risk factors were included (Table 4). Therefore, the FRAX model without BMD should not be used to predict OVCF with the current cutoff point, without a history of prior fracture included. As the FRAX model is currently being used as a guideline for considering a BMD test (i.e., the current recommendation threshold for BMD testing from 10-year FRAX-MOF for individuals age > 65 years is still 10 or more [2, 24]), the threshold should be modified for improved capability in OVCF diagnosis. Our sub-analysis also found that the optimum cutoff value to predict OVCF of FRAX-MOF without BMD was 3.9 (AUC 0.63, sensitivity of 85%, and specificity of 41%) and 0.7 for FRAX-HF without BMD (AUC 0.63, sensitivity of 87%, and specificity of 39%).

The OSTA index [15] and KKOS [16] were introduced to identify the population at risk for osteoporosis and to justify the BMD assessment rather than diagnose OVCF, as previously mentioned. However, Yang et al. reported that an OSTA index less than -1 could predict a new painful vertebral compression fracture, based on results from a self-report questionnaire among Chinese women, with yields of 66% sensitivity, 76% specificity, and an AUC of 0.812. Nevertheless, the same report did not mention asymptomatic OVCF [24]. Saetung et al. reported that the cutoff point of -1 in the OSTA index might predict new vertebral compression fracture with an AUC of 0.7, while the prevalence of OVCF in their study was only 7%, compared to the 29% prevalence of OVCF in the present study (Fig. 1) [25]. In this study, we used the cutoff point of less than -1 in the OSTA index and detected OVCF with an AUC of 0.59, sensitivity of 76%, and specificity of only 42% (Table 4). On the other hand, the KKOS model used age and weight to access the index of suspicion for osteoporosis based on the BMD, so such an index is justified for individuals whose BMD falls in the osteoporosis range (i.e., BMD T-score ≤—2.5). Currently, the occurrence of OVCF in individuals whose BMD falls in the osteopenic range has been reported [12, 17, 18]. The discriminating ability of KKOS to identify OVCF yielded a lower AUC than our final predictive model without BMD; AUC was only 0.58 with sensitivity of 73% and specificity of 44%. Therefore, the performance of both OSTA and KKOS to identify OVCF in clinical practice appears less satisfactory and, based on our study, might not be effective tools for identifying OVCF (Table 4). According to the present study without the BMD results, the OVCF screening should be performed for any individual woman over age 65 years who has an HHL of more than 1.5 cm.

The ISCD recommended a VFA test for those with a femoral neck T-score < -1 combined with one of the following criteria: (a) women aged ≥ 70 years, (b) women aged ≥ 80 years, (c) HHL over 4 cm, (d) self-reported but undocumented vertebral fracture, and (e) glucocorticoid intake of more than 5 mg of prednisolone or equivalent per day for more than 3 months [11, 12]. Based on our finding, the ISCD 2019 criteria revealed 60% sensitivity, 68% specificity, and an AUC of 0.64, compared to our predictive model, which yielded higher sensitivity, specificity, and AUC. This finding may be partly explained by the difference in cutoff values. The ISCD cutoff for height loss is 4 cm, which we found in our study to show only 15% sensitivity in ROC analysis compared to our cutoff criterion of 1.5 cm, used in this present study, which showed 54% sensitivity. Also with age, the cutoff value of ISCD is more than 70 years, which yields 57% sensitivity compared to the 65-year criterion in our present study yielding 77% sensitivity from the ROC analysis. Due to the criterion of height loss used in ISCD, the sensitivity to detect OVCF was lower than expected in our data. Our sub-analysis also revealed that height loss of more than 1.4 cm should be used as a criterion for OVCF screening in any individual woman with femoral neck T-score < -1. Our finding may be partially consistent with the result from the European Prospective Osteoporosis Study (EPOS), which revealed increments of RR of OVCF according to specific factors, such as RR of 1.32 per decade of advanced age and RR of 1.03 per 1 cm of height loss [26]. According to the EPOS recommendation, it would be necessary to perform the VFA test in women more than 65 years of age to prevent new vertebral fracture in the next 5 years by providing medical treatment. That recommendation agreed with our findings. Unfortunately, however, the EPOS model is still complex and difficult to use in daily practice.

The performance of the present model without BMD regarding age is illustrated in Table 5. The highest negative predictive value in the group with age < 65 years with an HHL of no more than 1.5 cm should be the specific exclusion criteria for the OVCF screening. The sensitivity, specificity, and AUC—when performed in 5-year age increments starting at age of 65 years—revealed little difference among groups and revealed high performance in age > 80 years. This finding could imply that advancing age has no effect on this predictive model, which is different from the other models (i.e., FRAX or OSTA). Regarding the findings in this present study, our recommendation is to consider a DXA test in any women with age more than 65 years and history of height loss more than 1.5 cm. Moreover, the VFA test should be included, when preformed DXA, in every individual woman with these criteria and having femoral neck T-score less than -1.7. Applying this trigger for conducting testing would have the benefit of early detection of OVCFs and would prevent subsequent vertebral and non-vertebral fractures by providing medical or surgical intervention.

Certain limitations in our study should be acknowledged. First, only glucocorticoid was included regardless the dosage; other potential risk factors were not included. The risk factors to estimate the OVCF may therefore not be precise. Second, the detection of vertebral fracture in non-fractured vertebral deformities in VFA may have some limitation. However, reasonable agreement between the VFA and lateral spine radiograph has been established in our institute [20]. Third, the criteria for OVCF screening in this study were obtained only from a risk factor analysis from a retrospective study, and the populations in this study were only female. Therefore, the application of our prediction model should be limited only to postmenopausal woman. Moreover, the cost-effectiveness or future prediction of OVCF was not a point of concern in this study.

In conclusion, early detection of OVCF is crucial to prevent subsequent fracture by identifying the need for early treatment of osteoporosis. However, diagnosing OVCF is challenging because almost all OVCF patients are asymptomatic. Regarding the findings from this present study, the BMD and vertebral fracture screening should be prescribed for any individual woman aged more than 65 years with an HLL more than 1.5 cm. A vertebral fracture assessment should be additionally examined for these females whose femoral neck T-scores are less than -1.7.

## Data Availability

The datasets used and analyzed during the current study are available from the corresponding author on reasonable request.

## Abbreviations

DXA: Dual-energy X-ray absorptiometry
OVCF: Osteoporotic vertebral compression fracture
VFA: Vertebral fracture assessment
BMD: Bone mass density
HHL: History of height loss
FRAX: Fracture risk assessment tool
ISCD: International Society for Clinical Densitometry
OSTA: Osteoporosis Self-Assessment Tool for Asians
EPOS: European Prospective Osteoporosis Study
TOPF: Thai Osteoporotic Foundation
KKOS: Khon Kaen Osteoporosis Study
PACS: Picture archiving and communication system
